# Lane Marking Detection and Reconstruction with Line-Scan Imaging Data

**DOI:** 10.3390/s18051635

**Published:** 2018-05-20

**Authors:** Lin Li, Wenting Luo, Kelvin C. P. Wang

**Affiliations:** 1 School of Transportation and Civil Engineering, Fujian Agriculture and Forestry University, Fuzhou 350002, China; 2 School of Civil and Environmental Engineering, Oklahoma State University, Stillwater, OK 74078, USA; kelvin.wang@okstate.edu

**Keywords:** laser sensor, line scan camera, lane marking detection, support vector machine (SVM), image binarization, lane marking reconstruction

## Abstract

Lane marking detection and localization are crucial for autonomous driving and lane-based pavement surveys. Numerous studies have been done to detect and locate lane markings with the purpose of advanced driver assistance systems, in which image data are usually captured by vision-based cameras. However, a limited number of studies have been done to identify lane markings using high-resolution laser images for road condition evaluation. In this study, the laser images are acquired with a digital highway data vehicle (DHDV). Subsequently, a novel methodology is presented for the automated lane marking identification and reconstruction, and is implemented in four phases: (1) binarization of the laser images with a new threshold method (multi-box segmentation based threshold method); (2) determination of candidate lane markings with closing operations and a marching square algorithm; (3) identification of true lane marking by eliminating false positives (FPs) using a linear support vector machine method; and (4) reconstruction of the damaged and dash lane marking segments to form a continuous lane marking based on the geometry features such as adjacent lane marking location and lane width. Finally, a case study is given to validate effects of the novel methodology. The findings indicate the new strategy is robust in image binarization and lane marking localization. This study would be beneficial in road lane-based pavement condition evaluation such as lane-based rutting measurement and crack classification.

## 1. Introduction

Road lane markings deteriorate from routine use, which can lead to unexpected traffic accidents for road users [1]. Usually, lane marking data can be acquired by various approaches, such as visual cameras, GPS sensors, radar sensors, and laser sensors [2,3,4]. Each acquisition method has its own advantages and limitations in different application fields. Previous studies indicate that lane marking data captured by visual cameras are widely used for autonomous driving navigation and traffic surveillance [2,5,6], based on which numerous efforts have been made to detect, locate, and track lane markings in the spatial domain. However, the study of lane marking detection and location for use in road condition evaluation is neglected.

Generally the detection and localization of lane markings can be roughly implemented in a three-step process: (1) extraction of the lane marking features though pre-processing operations (i.e., exposure correction and shadow removal…) [7,8,9]; (2) obtaining the location of true lane marking through a series of related process (i.e., thresholding, particle filtering, model fitting…) [10,11]; and (3) tracking the detected lane marking with different techniques (i.e., temporal consistency, position consistency, Hough transform...) [12,13,14]. However, unexpected challenges always appear in lane marking detection and localization due to various interferences such as illumination conditions (occlusion, night time…), camera location and orientation, environmental factors (i.e., foggy days, cloudy and rainy days…), the appearance of the lane markings, the type of road, and so on [2]. To deal with the abovementioned problems, numerous vision-based lane marking detection and localization algorithms have been proposed, which for structured roads can be roughly grouped into two categories: feature-based methods and model-based techniques [6,15,16,17,18].

Feature-based methods identify road lane markings with low-level features such as line edges and colors [19]. Traditional edge-based segmentation methods such as the watershed transformation [20], the OTSU segmentation method [21], and Canny edge detectors [22] are used to identify lane markings. However, these traditional methods are susceptible to the effects of occlusions and intensity noise, and thus produce unsatisfactory identification results. Color representation is a widely used technique in image processing, which captures the feature information of lane markings in several color spaces (i.e., RGB, HSI and XYZ) [23,24,25,26,27]. The authors in [28] compared the effectiveness of color representation in HSI and RGB space, and then developed an adaptive method for lane marking identification in HSI color space. Although HSI-based color representation can alleviate the influence of brightness changes, it tends to confuse true targets with noises when the color information is similar. Moreover, color representation cannot comprehensively disclose lane marking features so that its use should be in combination with other non-color features such as lane edges or corners, painted lines, etc. [29,30,31]. The authors in [32] analyzed low-level features by using an adaptive segmentation method, and then an efficient line segment detector was proposed for lane marking detection. However, one explicit limitation exists for feature-based methods, that is, it requires the well-painted road or strong lane edges, therefore, it may suffer from background noises.

Model-based methods use a few parameters or templates to represent the lines by assuming straight lines or parabolic curves [6,33]. These techniques are more robust in noise removal, probably due to their high-level processing instead of pixel-based processing. Deformable template models that describe road edges in terms of their curvature, orientation, and offset are proposed to locate the lane boundaries [34,35]. These models are deformable so that they can best fit or match the underlying intensity variation [36], which enables them to detect lane markings in situations with shadows and broken segments since thresholding of the intensity information is ignored. A lane detection and tracking algorithm was initiated based on B-snakes [11]. This method can describe a lane through a wide range of lane structures since this model can form an arbitrary shape by a set of control points. Linear-parabolic lane models are proposed for lane departure warning systems, in which the linear function and quadratic function are used to model the lane markings in the near field and far field, respectively [33,37]. Hough Transform (HT) and its variants (e.g., improved HT, randomized HT, hierarchical HT) are widely used for straight or curved lane marking detection [2,38,39,40,41]. However, one primary limitation of this method is how to model arbitrary road shape. Furthermore, model parameters’ setting and computation are an iterative trial-and-error process, which requires both human expertise and labor.

Note that the abovementioned approaches may perform well for the color images captured by an on-board camera of a vehicle and fulfill their application in driving assistance systems. However, studies on lane-based infrastructure performance assessment using 2D laser images are neglected.

Although lots of efforts have been made on pavement distress identification and rutting measurement in the past several decades [42], road lane boundaries cannot be accurately positioned, thus resulting in the inaccuracy of lane-based distress classification and performance assessments. To implement lane-based distress evaluation (i.e., pavement cracks, rutting measurement) using 2D laser images, a robust lane detection and localization approach is presented in this study. Firstly, 2D laser image data are collected by the Digital Highway Data Vehicle (DHDV) which is a real-time multi-functional system for roadway data acquisition, and then sigmoid correction method is used for background noise removal and contrast enhancement. Subsequently a new thresholding strategy is proposed to binarize laser images, based on which the pixel-based contour traversal method is developed to produce the contour boxes used as basic elements for lane marking identification. Thirdly, a Linear Support Vector Machine (LSVM) is introduced to determine proper vector weights and bias to discriminate true lane markings from noises based on contour box attributes. Finally, true lane markings along the traveling direction can be continuously reconstructed using the geometry information of the previous and current frames or images. To validate effects of the new methodology on lane marking detection and localization, a 2.286 km-long pavement section (including 1000 laser images) is chosen as a test bed. The performance of the new methodology is evaluated using Precision-Curve (PR) analysis. Results indicate the new methodology is robust and reliable in lane marking detection and localization for laser images. This study would be beneficial in continuous measurement and evaluation of lane-based pavement distress for project- and network-level pavement survey.

## 2. Data Acquisition System

**Figure 1:**
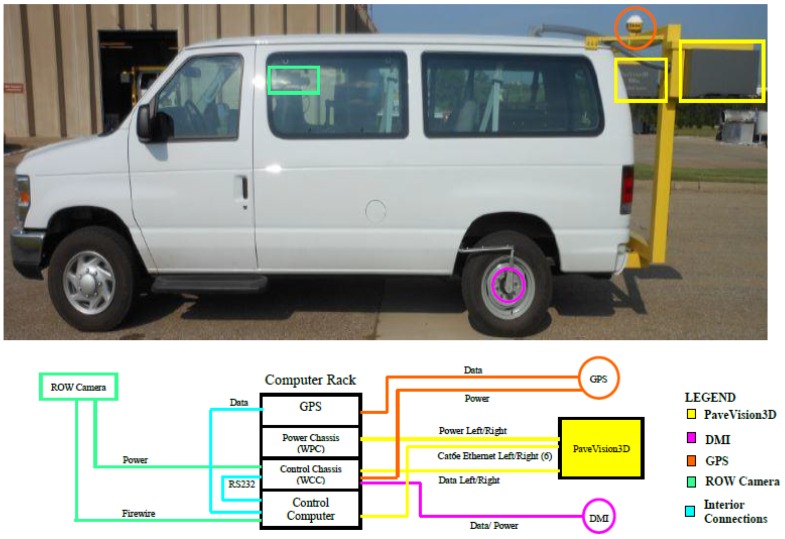
Generic DHDV subsystem overview.

The DHDV is a real-time multi-functional system for roadway data acquisition and analysis, particularly for pavement surface distress survey, roughness- and safety-related pavement performance evaluation [42]. The PaveVision3D Ultra (3D Ultra for short) system is the latest imaging sensor technology that enables one to acquire both 2D and 3D laser imaging data from pavement surfaces through two separate left and right sensors. The system is made up of eight high resolution cameras and two sets of lasers and is capable of constructing 4096 × 2048 images of full-lane width pavement surface with complete and continuous coverage. The subsystems of the DHDV vehicle include one 8-core computer, a Waylink Power Chassis (WPC), a WayLink Control Chassis (WCC), a differential GPS receiver or Inertial Measuring Unit (IMU), a Distance Measuring Instrument (DMI), and laser imaging sensors, as illustrated in Figure 1.

**Figure 2:**
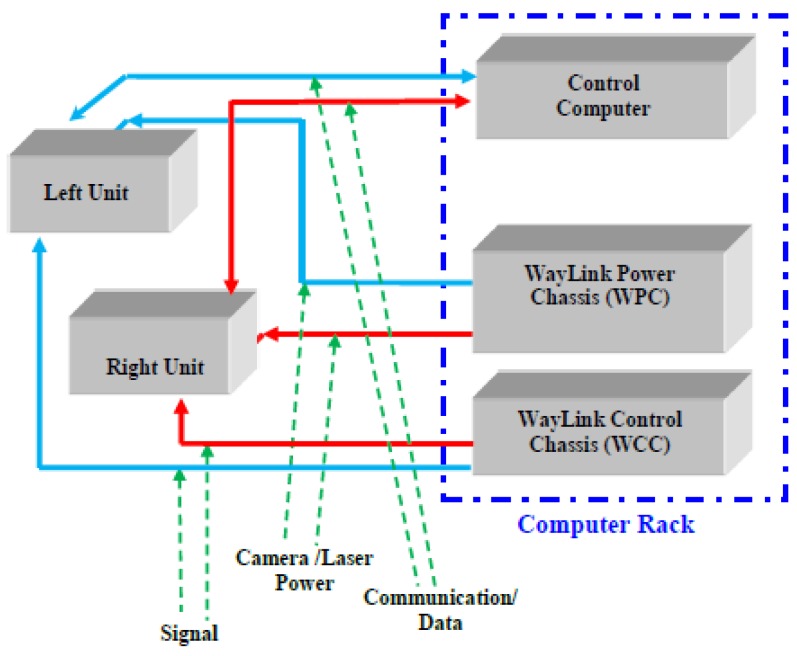
Line scan camera wiring diagram.

With the high-power line laser projection system and custom optic filters, the DHDV can work at highway speeds during daytime and nighttime and maintain image quality and consistency. That means the images are shadow-free at any time of the day. Figure 2 demonstrates the wiring of the cameras and lasers to the computer rack inside the vehicle. The cameras and lasers are powered by WPC and triggered by the WCC. The WCC connects to the Control Computer. The cameras are mounted on an aluminum alignment frame spaced equidistant from previously calibrated readings. The cameras and lasers reside inside two water-tight, aluminum containers, which are mounted on the external DHDV frame. The calibrated spacing of the cameras ensures that captured laser images can cover four-meter-wide pavements. The height of the sensors has been specifically designed for cameras to accurate capture data within the laser illumination ranges.

**Figure 3:**
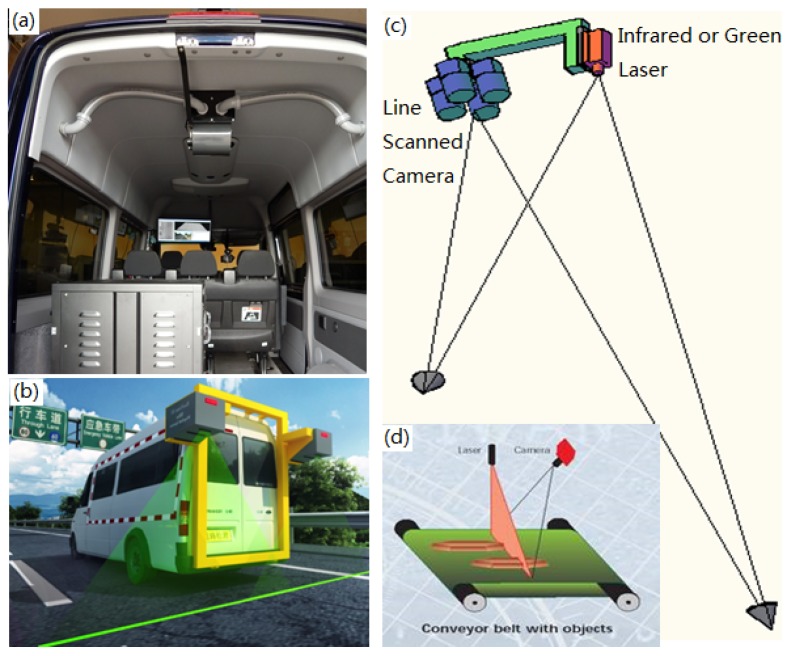
Photos of (**a**) DHDV interior appearance (**b**) DHDV rear view with PaveVision3D sensors; and (**c**,**d**) Pavevision3D working principle.

Figure 3a shows the interior appearance. Figure 3b shows rear view of the working DHDV equipped with the 3D Ultra technology. The camera and laser working principle are depicted in Figure 3c,d. By illuminating a surface using a line laser and shooting both 2D and 3D images using the corresponding cameras, the surface intensity and height variation information can be captured, in which surface height information is calculated from the distance from the camera to pavement based on the laser points (termed as the triangulation principle).

From Figure 3b, it can be observed that the width of laser images acquired from DHDV is more than the width of highway lanes (e.g., 3.66 m in United States) [43]. Accordingly, the exact detection and location of road lane marking are significant for lane-based pavement distress measurement and evaluation.

## 3. Methodologies

**Figure 4:**
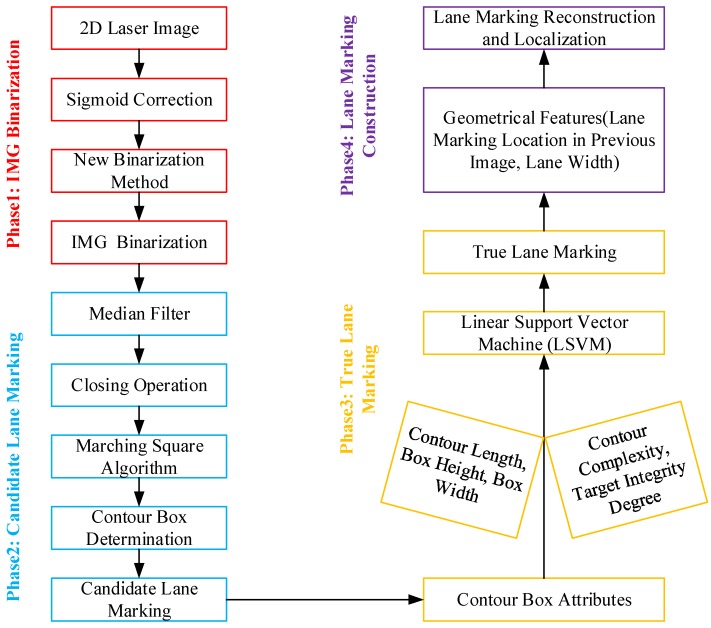
Schematic of the new methodology for automated identification and localization of lane marking.

To achieve this objective, a series of image processing techniques are presented in this paper, which can be classified into four phases, as illustrated in Figure 4. The first phase is to binarize 2D the laser images with sigmoid correction and a new threshold method; the second phase is to delineate all contour boxes or candidate lane markings based on closing operation and marching square algorithm; the third phase is to separate out true lane marking from candidate lane marking using LSVM based on contour box attributes; and the last phase is to reconstruct broken and inconsecutive segments and form the continuous lane marking along traveling direction. As a consequence, the exact location of lane marking of the entire pavement section can be obtained, and the lane-based pavement distress survey can be performed.

### 3.1. Image Binarization

During laser image data collection, some unexpected errors or intensity noises (i.e., whitening strips in travel direction) might be produced due to the presence of non-uniformity of laser intensity, lens distortion, physical installation locations of cameras. Therefore, maximally suppressing effects of noises on target detection is critical for the laser image binarization.

#### 3.1.1. Data Preprocessing

To maximally suppress background noises and enhance the contrast between targets (lane marking) and background noises, histogram equalization and sigmoid correction are introduced, in which the method that produces better pre-processing results would be used in this paper.

Histogram equalization is a widely used method in image contrast enhancement [44]. The basic idea behind this method is to redistribute all pixel values to be as close as possible to a specified desired histogram. Its mathematical description can be given in (1) and (2):

(1) $$P_{r}(r^{k})=n^{k}/n$$

(2) $$T(r^{i})=r\times \sum_{i=0}^{k-1}P_{r}(r^{i})$$

where *r* represents the grayscale range of 2D image data (in this case *r* = 255), *P*_r_(*r^k^*) stands for the frequency of grayscale value of *r^k^*; *n^k^* is the number of grayscale value of *r^k^*; *n* is the total of all pixels; *T*(*r^i^*) represents the new grayscale value for the grayscale of *r^i^*.

Sigmoid correction method uses a continuous non-linear function to transform the normalized pixel values of input images to the pixel values of output images [45], and its mathematical equation can be described in (3):

(3) $$I_{out}=\frac{1}{1+e^{gain\times (cutoff-I_{in})}}$$

where *I*_in_ and *I*_out_ respectively represent the normalized pixel values of input and output images; *gain* is the multiplier in exponential’s power of sigmoid function; *cutoff* is the shift value of the characteristic curve in horizontal direction. Note that both *gain* and *cutoff* should be properly initialized before use.

**Figure 5:**
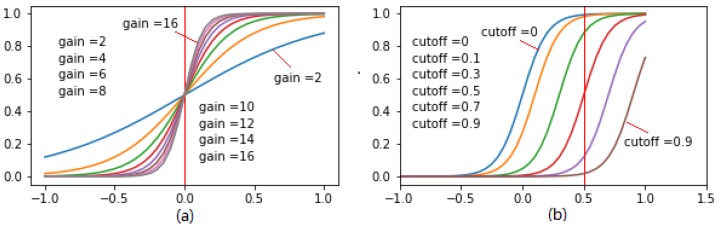
Sigmoid functions of (**a**) with different gains (cutoff = 0); (**b**) with different cutoffs (gain = 10).

Note that sigmoid function is ‘S’ shaped, as shown in Figure 5. Figure 5a shows the transform trend ranged at [−0.5, 0.5] decreases sharply with the decrease of gain, and it becomes approximately linear when the gain variable equals to 2. The cutoff variable shifts the curve characteristics in the horizontal direction, as shown in Figure 5b. In this study, the gain of 10 and the cutoff of 0.5 are chosen after several rounds of trial and error.

**Figure 6:**
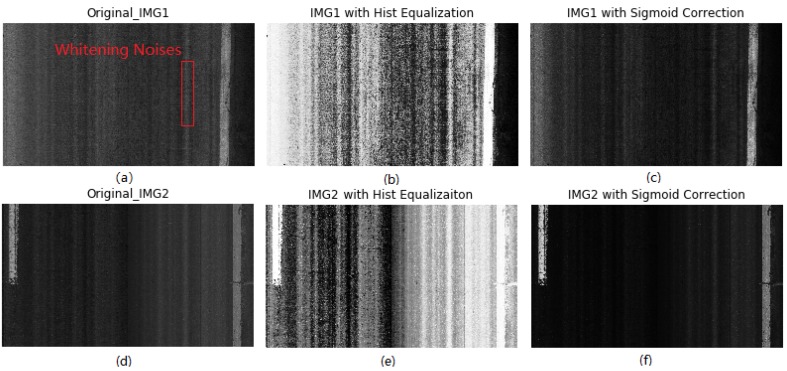
Photographs of noise removal: (**a**) raw image1 with whitening noises; (**b**) IMG1 with histogram equalization; (**c**) IMG1 with sigmoid correction; (**d**) Original_IMG2; (**e**) IMG2 with histogram equalization; (**f**) IMG2 with sigmoid correction.

To examine the effects of the two techniques on background noise removal and contrast enhancement, two laser images (Original_IMG1 and Original_IMG2) are chosen as test specimens, as shown in Figure 6a,d. It can be observed that both images contain whitening strips or noises, as red rectangle marks. Subsequently, the two methods are respectively applied on the two images for noise removal. Figure 6b,c,e,f represent the pre-processing results of Original_IMG1 and Original_IMG2 with the two different techniques. Note that the sigmoid correction method has better performance in separating background from foreground (lane marking) than histogram equalization. For the sigmoid correction method, the background pixels become much darker than that in the original images, that is, the influences of background noises on laser image binarization are greatly suppressed. Meanwhile, intensities of foreground pixels are increased, that is, lane marking would be easier to be identified out in the process of image binarization. Therefore, the sigmoid correction is chosen and used for background noise removal and contrast enhancement.

#### 3.1.2. New Binarization Method

Once noise removal and contrast enhancement are accomplished, the following task is image binarization. In this study, two methods, namely OTSU method and minimum threshold method are examined for this purpose. The OTSU method is a clustering-based image thresholding method [46]. The algorithm assumes that the image contains two classes of pixels following bi-modal histogram (foreground pixels and background pixels), and then it calculates the optimum threshold separating the two classes so that their combined spread (intra-class variance) is minimal. The mathematical description is given in (4)–(6):

(4) $$\sigma_{\mathrm{intra}}^{2}=\omega_{0}(t)\omega_{1}(t){\left[\mu_{0}(t)-\mu_{1}(t)\right]}^{2}$$

(5) $$\omega_{0}(t)+\omega_{1}(t)=1$$

(6) $$\omega_{0}(t)\mu_{0}(t)+\omega_{1}(t)\mu_{1}(t)=\mu (t)$$

where weights *ω*_0_ and *ω*_1_ are the probabilities of the two classes separated by a threshold *t*; *σ*^2^_intra_ are variances of these two classes, *μ*_0_ and *μ*_1_ respectively represent the means of these two classes.

The minimum threshold method [47,48] is suitable for binarizing images with two spikes or maxima so that the algorithm requires keep calculating and smoothing the histogram of the input image until there are only two maxima. Subsequently the threshold can be determined by the minimum value between the two maxima. However, in fields the laser image may not have the two maxima, and thus the threshold method would fail in image processing. To deal with this problem, the minimum thresholding method is modified to adapt the binarization of the image with one spike, and its mathematical expression is given in (7):

(7) $$T=\left\{ \begin{array}{l} f(h_{1}+T_{m})/2\\ f(\min(h_{i}) \end{array}\right.,\;h_{i}\in (h_{1},h_{2})$$

where *T* is the minimum threshold; *h*_1_ and *h*_2_ represents the two maxima of the histograms of the input image; *T*_m_ is the maxima intensity of input image; *f* is used to calculate the threshold.

**Figure 7:**
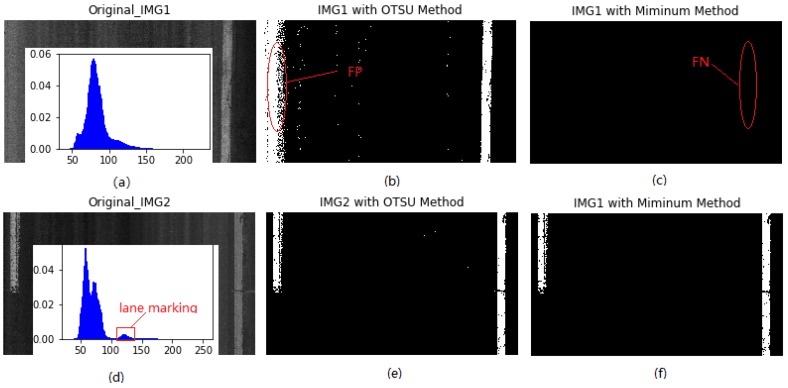
Binarization results: (**a**) IMG1 and its histogram; (**b**) binarized IMG1 with OTSU method; (**c**) binarized IMG1 with minimum method; (**d**) IMG2 and its histogram; (**e**) binarized IMG2 with OTSU method; (**f**) binarized IMG2 with minimum method.

Figure 7a,d show two 2D laser images and their histogram distribution, respectively. Note that IMG2 has the two spikes, and both methods produce excellent binarization results for IMG2 since the histogram distribution of IMG2 has two maxima. It can be found that the two methods perform well in binarization for laser images that have two spikes in their histogram distribution, based on which the optimal threshold can be determined, as shown in Figure 7e,f. For IMG1, however, both methods produce the poor binarization results since it only has one single maximum. In this case, the OTSU method produces a false positive (FP) result, while the modified minimum threshold method produces a false negative (FN) result, as the red circles show in Figure 7b,c, respectively. It can be concluded that both methods fail to binarize the laser image that has one single spike in its histogram distribution.

**Figure 8:**
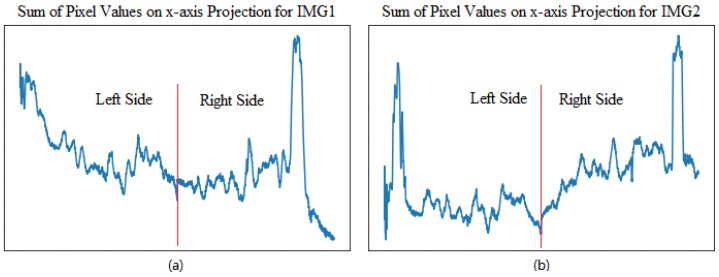
Pixel intensity sum’s projection on x-axis for: (**a**) IMG1; and (**b**) IMG2.

To investigate the cause why the two methods fail in Original_IMG1 binarization, the sum of pixel intensity in the vertical direction is projected onto the x-axis for IMG1 and IMG2, as plotted in Figure 8a,b, respectively. In this study one laser image is obtained by merging pixel data derived from the left and right cameras. Note that IMG2 has a strong contrast between background and foreground pixels for both sides of the laser image, that is, the foreground and background are apparent and easily distinguished, as shown in Figure 8b. For the left-sided lane marking of IMG1 in Figure 8a, however, a low contrast is observed, indicating the background and foreground are indistinct and thus are cumbersome to separate out. To deal with the issue that may be caused by the non-uniformity of laser intensity, the multi-box segmentation-based threshold method is proposed.

The basic idea behind the new binarization method is to divide one laser image into multiple small segmentation regions, and subsequently the threshold operation is performed on each individual segmentation region. Its implementation can be elaborated below: (1) partition 2D laser image into the left and right sides (i.e., IMG_L and IMG_R) since each 2D laser image is made of two components derived from two different cameras mounted on DHDV, and thus the better binarization result might be obtained once the left and right sides are separated out; (2) divide both left and right sides of images into multiple small regions (i.e., IMG_L_1, …, IMG_L_N, N is the number of small segmentation regions for left side) along traveling direction, and the corresponding threshold can be obtained; (3) recalculate the new threshold for each small region based on minimum square error method; and (4) reconstruct the binarized images by merging all small segmentation boxes in sequence. The new threshold for each segmentation box can be calculated using (8)–(10):

(8) $${\hat{\beta }}_{0}=\frac{\sum_{i=1}^{n}X_{i}^{2}\sum_{i=1}^{n}Y_{i}-\sum_{i=1}^{n}X_{i}\sum_{i=1}^{n}X_{i}Y_{i}}{n\sum_{i=1}^{n}X_{i}^{2}-{\left(\sum_{i=1}^{n}X_{i}\right)}^{2}}$$

(9) $${\hat{\beta }}_{1}=\frac{\sum_{i=1}^{n}X_{i}Y_{i}-\sum_{i=1}^{n}X_{i}\sum_{i=1}^{n}Y_{i}}{n\sum_{i=1}^{n}X_{i}^{2}-{\left(\sum_{i=1}^{n}X_{i}\right)}^{2}}$$

(10) $$Y_{Newi}={\hat{\beta }}_{0}+{\hat{\beta }}_{1}X_{i}$$

where *X_i_*, *Y_i_* represent the *i*-th small segmentation region in sequence and its corresponding threshold, respectively; *n* is the number of small segmentation regions for each side of image; ${\hat{\beta }}_{0},\;{\hat{\beta }}_{1}$ refer to the regression coefficients of the ordinary least square errors. $Y_{New_{i}}\;$is the new threshold for the segmentation region *i*.

**Figure 9:**
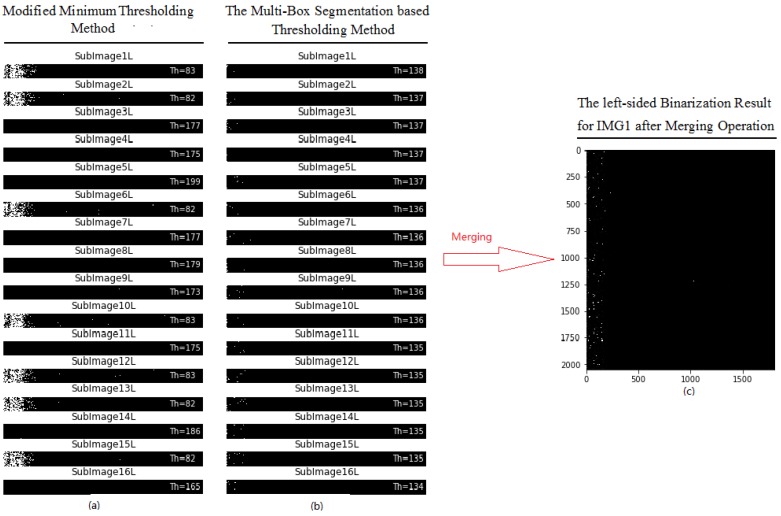
Binarization with the new method: (**a**) segmentation regions with their thresholds; (**b**) segmentation regions with new thresholds; and (**c**) image binarization result with new threshold.

Figure 9 shows the working principle of the new binarization method. Firstly, the left side of IMG1 is partitioned into 16 small segmentation regions (*X_i_*), and the modified minimum threshold method is used on each small region to calculate thresholds (*Y_i_*). The calculated threshold for each small region are shown on Figure 9a. Note that the different segmentation regions have different thresholds, and the two adjacent regions may even have a sharp variation in threshold (i.e., region IDs 2 and 3). The large variation in threshold may be caused by two underlying reasons: (1) the inconsistency or ununiform of pixel intensity of images, and (2) the drawback or limitation of the threshold method.

To deal with this issue, the minimum square error method is used to recalculate thresholds for each segmentation region based on the pre-calculated thresholds (*Y_i_*) from 16 segmentation regions. Once the coefficients of linear regression model are obtained, the new threshold ($Y_{New_{i}}$) for each segmentation region can be recalculated, as shown in Figure 9b. Note that the new thresholds between the adjacent segmentation regions display smooth changes, with a threshold value of approximately 137. Finally, the left side of IMG1 can be reconstructed by merging all small regions that have been binarized with the new threshold, as shown in Figure 9c.

**Figure 10:**
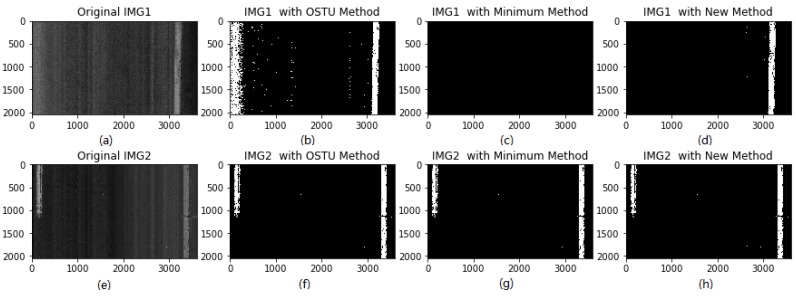
Comparison of binarization results: (**a**–**d**) IMG1 and its corresponding threshold methods; and (**e**–**h**) IMG2 and its corresponding threshold methods.

Figure 10a–h show the effects of the new binarization method, OSTU method, and the modified minimum threshold method on laser images. It can be found that the new threshold method produces the best binarization results. For IMG2, all three methods can produce decent binarization results for lane markings, except for several whitened spots. For IMG1, the OSTU threshold method produces a false positive binarization result, and the modified minimum threshold method produces a false negative binarization results. The new threshold method produces an excellent binarization result for IMG1, and the true positive and true negative binarization results are produced. Therefore, in this paper, the new method, namely the multi-box segmentation-based traversal method, is used for 2D laser image binarization.

### 3.2. Candiate Lane Marking

Once 2D laser images are binarized with the new threshold method, the following task is to determine whether any whitened strips in binary images belong to lane markings or not. Firstly, a median filter is employed to eliminate the discrete spots or small blobs that are produced in binarization. Usually the discrete spots or small blobs can be assumed as fake targets and should be eliminated. Secondly, morphological closing operation and marching square algorithm are used to obtain the contour of each whitening strip or blob, and then contour box-based method is proposed to frame each whitening strip or blob. In this study, each contour box is considered as a candidate lane marking, and is taken as a basic element for the true lane marking identification.

#### 3.2.1. Closing Operation

Due to the existence of noises such as the whitening aggregates and others, the binarized images may contain some discrete pixels or spots. To eliminate the influence of discrete spots on true lane marking identification, a median filter is employed to remove the discrete none-zero pixels.

Pavement distress such as cracking or potholes will appear during pavement aging. As a result, one entire lane marking or whitening strips may be broken into several segments by cracks, which results in extra difficulties in true lane marking identification. To deal with this issue, the morphological closing operation is used to stitch the separated whitening strips with gaps in between and produce one well-connected strip, and simultaneously the discrete white pixels are eliminated. The morphological closing operation is defined as a dilation followed by an erosion [49]. The closing operation can remove small bright spots and patch small dark cracks in lane markings. Erosion removes the non-zero pixels from object boundaries to shrink the boundaries, while the dilation operation adds binary pixels with non-zero values to the boundaries of objects in an image to fill the gaps and enlarge boundaries [42]. The number of pixels added or removed from the objects in an image depends on the size and shape of the structuring element used to process the image. The structuring element defines the neighborhood of the pixel of interest. In this study, the structuring element with a size of 15 × 15-pixel matrix is used after several trials and errors.

**Figure 11:**
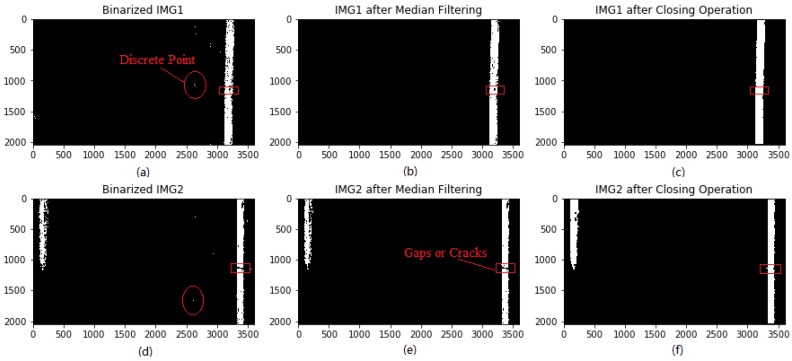
Photographs of morphological operation: (**a**,**d**) images after binarization; (**b**,**e**) binarized images after median filtering; (**c**,**f**) binarized images after closing operation.

In Figure 11a,d, the discrete spots and lane marking gaps are marked using red circles and rectangles, respectively. Firstly, median filtering is used to remove the discrete spots, as shown in Figure 11b,e. It can be observed that the discrete spots inside circles are totally removed. Subsequently, closing operations is employed to stitch lane marking with gaps in between and produces one independent and well-connected strip. From Figure 11c,f, it can be observed that the gap or crack inside rectangles are fully filled up. Accordingly, both median filter and closing operation are robust in eliminating discrete spots and patching up lane marking gaps, which are crucial for removing fake targets and determining candidate lane markings.

#### 3.2.2. Marching Square Algorithm

**Figure 12:**
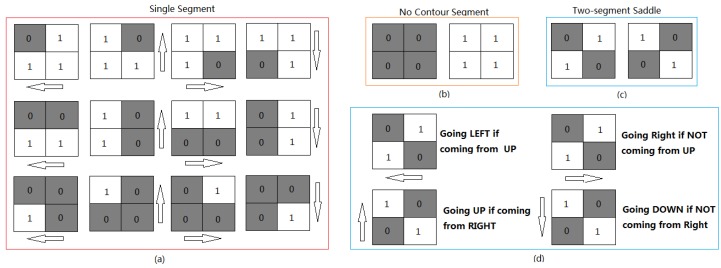
Photographs of (**a**) 12-moving direction for single segment; (**b**) no contour segment; (**c**) two-segment saddle; and (**d**) the 4-moving direction for the two-segment saddle.

All candidate lane markings should be found before true lane marking identification. To achieve this goal, a marching square algorithm is introduced to generate the contour of the segmentation region for a two-dimensional image [50]. For one binary image, every 2 × 2 block of pixels (see Figure 12) forms a contouring box or cell, so the entire image can be represented by numerous contouring boxes. The important thing in marching square algorithm is the “sense of direction”. The moving direction you head are with respect to your current positioning, which depends on the way you entered the pixel you are standing on. Therefore, it’s important to keep track of your current orientation.

The algorithm can be described as follows: (1) assume that you stand on the start pixel of one image binary; (2) observe the up, left, and up left pixel values, and then pick next moving direction based on Figure 12. For ‘single segment’ case, it easy to determine the next moving direction by matching the right contouring box, as shown in Figure 12a. For two-segment saddle (see Figure 12c), each contouring box can be divided into two states and their moving direction, as given in Figure 12d; and (3) keep moving until you get back the start position, and pixels you walked over would be the contour of the pattern.

**Figure 13:**
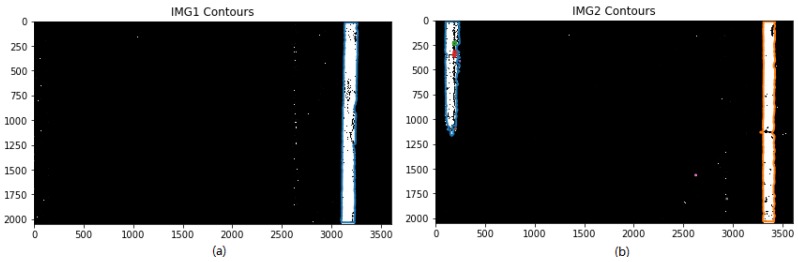
Photographs after the use of marching square algorithm: (**a**) one contour box for IMG1; and (**b**) eight contour boxes for IMG2 (as different colors show).

The marching square algorithm is used on binary images (i.e., IMG1 and IMG2) that have been pre-processed with median and closing operations, and then the contours of candidate lane marking can be obtained, as shown in Figure 13, which shows that IMG1 only has one contour box, indicating only one candidate lane marking needs to be judged whether it belongs to true lane marking or not. Figure 13b shows there are eight contour boxes for IMG2, indicating there are eight candidate lane markings that need to be validated which one or two belong to true lane marking or not.

### 3.3. True Lane Marking

Contour box attributes (i.e., box width, box height, contour complexity, contour length, and target integrity degree) for each candidate lane marking are calculated along with contour box determination. They are stored into arrays and used for separating true lane marking from noises. In this study contour box attributes are defined below:

#### 3.3.1. Contour Box Attributes

Contour box width and height are pixel differences between the minimum and maximum coordinates of contouring box in *x*-axis and *y*-axis, respectively. Contour length is the number of pixels that comprise object contours. Contour complexity is calculated by the contour length divided by the perimeter of boundary box. Contour complexity should approximate to 1 if the candidate lane marking belongs to true lane marking. Target integrity degree *I_t_* equals to one minus the root of square sum of gradients regions ∇x and ∇y, which is used to help judge whether candidate lane marking belongs to true lane marking or not. The target integrity degree is close to one if the candidate lane marking is true lane marking. The mathematical description of target integrity degree is given in (11):

(11) It=(1−(∂z∂x)2+(∂z∂y)2)×100%

where *I_t_* represents the target (lane marking) integrity degree; *z* represents the binary values at point (x,y); ∂*z*/∂*x* denotes the first-derivative of binary image in the *x* direction; ∂*z*/∂*y* denotes the first-derivative of binary image in the *y* direction.

**Figure 14:**
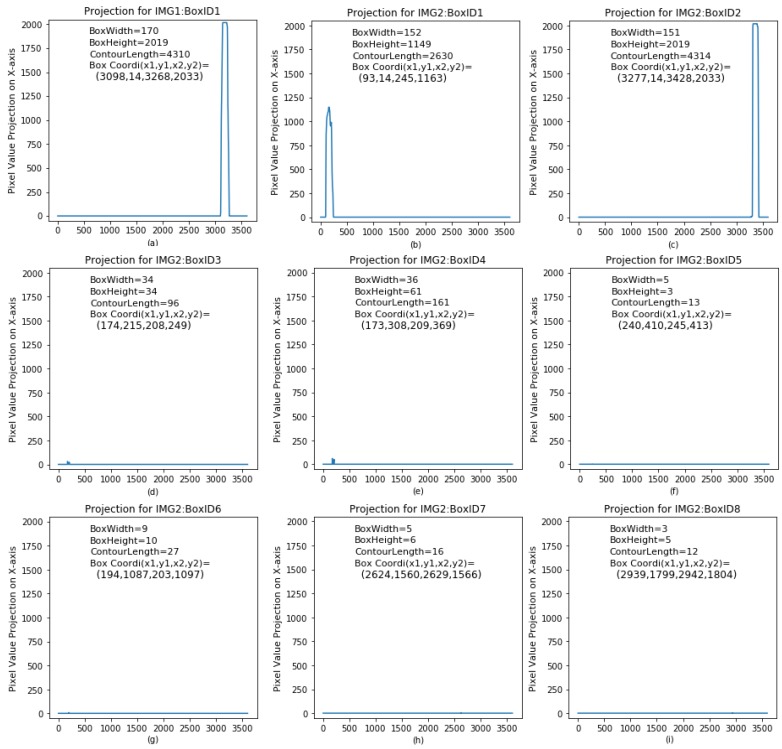
Photographs of the binary pixel’s projection on *X*-axis for each contour box: (**a**) BOXID1 for IMG1; and (**b**–**i**) BOXID1 to 8 for IMG2.

In general, each candidate lane marking belongs to either a true lane marking or noises, which depends on four contour box attributes: contour box width, contour box height, contour complexity, and target integrity degree. Table 1 shows contour box attributes of each candidate lane marking. In addition, the sum of pixel intensity for each contour box is projected onto the *X*-axis, as shown in Figure 14. IMG1 has one single contour box namely BoxID1, and its binary projection on *X*-axis is plotted in Figure 14a. IMG2 has eight contour boxes namely from BoxID1 to BoxID8, and their binary projections on *X*-axis are plotted in Figure 14b–i, respectively. It is apparent that IMG1 has one true lane marking based on its pixel projection on *X*-axis. IMG2 has a pair of lane marking, based on its pixel projection on *X*-axis in Figure 14b,c. For other contour boxes, their binary projections on *X*-axis are not apparent and can be negligible, and thus these contour boxes or candidate lane markings do not belong to true lane marking.

In summary, it can be preliminarily concluded that IMG1_ID1, IMG2_ID1 and IMG_ID2 belong to true lane markings based on their contour box attributes and binary projections on the *X*-axis. To efficiently separate out true lane marking from fake targets, linear support vector machine is presented in this study.

#### 3.3.2. Linear Support Vector Machine (LSVM)

A Linear Support Vector Machine (LSVM) is used to separate out true lane markings from candidate lane markings based on three variables since contour box height may be very low in laser images due to the presence of dash lane markings. SVM model is a representation of the samples as points in space and is mapped so that the samples of the separate categories are divided by a clear gap that is as wide as possible [51,52]. Typically, this clear gap is defined as the hyper plane, and the distance between hyper plane and the corresponding support vectors equals to 1/||w||.

Once the hyper plane is located, the new sample is then mapped into that same space and predicted to belong to a classification based on which side of the hyperplane they fall. The key of the LSVM is to determine the vector weights *W* and the bias *b* of the hyperplane *g*(*X*). The hyperplane can be mathematically expressed using (12):

(12) $$g(X)=W^{T}X+b$$

where *X* = [*x_w_*,*x_c_*,*x_t_*] is a 3-dimentional vector (inputs), *x_w_*,*x_c_*,*x_t_* represent the contour box width, contour complexity and target integrity degree, respectively; *W* = [*w_w_*,*w_c_*,*w_t_*] are three vector weights or the normal vector to hyper plane; *b* is the bias of the hyperplane.

To use the vector weight *W* and the bias *b* to separate out true lane marking from candidate lane marking, they should be computed first based on the labeled training data [*X^p^*,*δ^p^*]]. *p* represents the training sample number. *Y* is either 1 or −1, denoting the class to which the input vector *X* belongs, if the predicted *g*(*X*) is larger than zero, the input vector belongs to true lane marking, otherwise it belongs to noise box, which can be described using (13):

(13) $$Y^{p}(W^{T}X^{p})+b\geq 1$$

To calculate the maximum-margin hyper plane, the cost function $\Phi (W)=\frac{1}{2}W^{T}W$ is introduced and minimized. Equation (13) is one equality constraint of cost function. It is well known that the Lagrange function is widely used to deal with the optimization problem that finds the local minima or maxima of a function. In this study it is introduced to find the optimal solutions of *W*_0_ and *b*_0_, and its mathematical expression is (14):

(14) $$L(W,b,\alpha )=\frac{1}{2}W^{T}W-\sum_{p=1}^{P}\alpha_{p}[Y^{p}(W^{T}X^{p}+b)-1]$$

where L(W,b,α) is the Lagrange function or expression; *α_p_* is the Lagrange multiplier, and its value is no less than 0.

To minimize Lagrange function, the calculation of partial derivatives of *L*(*W*,*b*,*α*) with respect to vector weights and bias can be mathematically expressed in (15) and (16). Subsequently, the calculated vector weights are given in (17), and one equality constraint is obtained and given in (18):

(15) $$\frac{\partial L(W,b,\alpha )}{\partial w}=0$$

(16) $$\frac{\partial L(W,b,\alpha )}{\partial b}=0$$

(17) $$W=\sum_{p=1}^{P}\alpha_{p}Y^{p}X^{p}$$

(18) $$\sum_{p=1}^{P}\alpha_{p}Y^{p}=0$$

Using (17) to replace *W* in (14), the Lagrange function can be rewritten as (19). According to the Kuhn Tucker theory [53], the optimal solution for (19) can be deduced and rewritten as (20):

(19) $$L(W,b,\alpha )=\sum_{p=1}^{P}\alpha_{p}-\frac{1}{2}\sum_{p=1}^{P}\sum_{j=1}^{P}\alpha_{p}\alpha_{j}Y^{p}Y^{j}{\left(X^{p}\right)}^{T}X^{j}$$

(20) $$\alpha_{p}[Y^{p}(W^{T}X^{p}+b)-1]=0,\;\alpha_{p}>0$$

Assume the optimal Lagrange multiplier is {*α*_0*p*_, *α*_1*p*_,Λ,*α*_0*p*_}, the optimal weight vector can be calculated and rewritten as (21), and the optimal bias can be calculated using (22). Once *W*_0_ and *b*_0_ are calculated, the hyperplane coefficients can be determined accordingly:

(21) $$W_{0}=\sum_{p=1}^{P}\alpha_{0p}Y^{p}X^{p}=\sum_{ASV}\alpha_{0s}Y^{s}X^{s}$$

(22) $$b_{0}=1-W_{0}{}^{T}X^{s}$$

where *X^s^* is the support vector sample; *ASV* is defined as all support vectors; *α*_0*s*_ is the Lagrange multiplier of the support vector sample *X^s^*; *Y^s^* is the classification label for the support vector sample *X^s^*.

**Figure 15:**
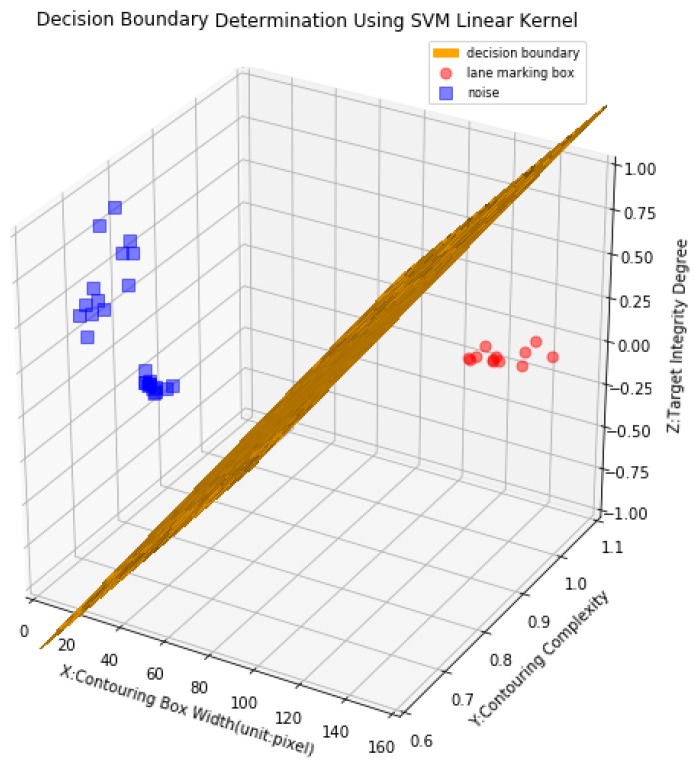
Illustration of hyperplane to separate true lane marking box from noise box.

Eight continuous 2D laser images are chosen to illustrate how LSVM works. 38 contour boxes (*p* = 38) and their corresponding contour box attributes are obtained via a series of image processing operations. Subsequently the LSVM model is employed to fit sample features *X* with classification labels *Y*. The weight vector *W*_0_ = [*w*_0w_,*w*_0*c*_,*w*_0t_] = [2.38092890 × 10^−2^, 7.31285305 × 10^−5^, −1.41721958 × 10^−5^] and the bias *b*_0_ = −1.92861422 are trained. Finally, the hyperplane or decision boundary can be plotted as seen in Figure 15.

As a result, the category that the contouring box belongs to can be determined based on (23). If the sign of the function *f*(*X*) is positive, the contouring box is a true lane marking box, otherwise it is a noise box:

(23) $$f(X)=\mathrm{sgn}(W_{0}^{T}+b_{0})$$

### 3.4. Lane Marking Reconstruction

**Figure 16:**
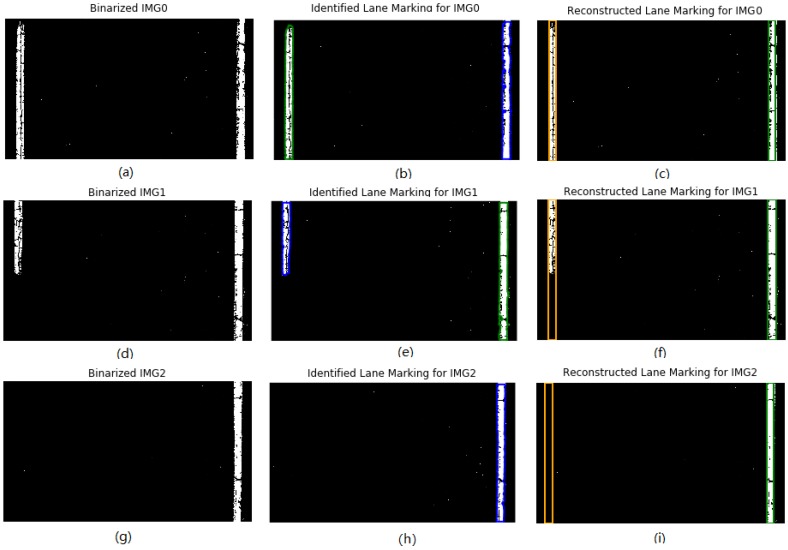
Binarization, Identification, and Reconstruction for solid and dash lane markings: (**a**–**c**) for IMG0; (**d**–**f**) for IMG1; and (**g**–**i**) for IMG2.

In this study the 2D laser image contains either one or a pair of lane markings, as shown in Figure 16a,d,g. For images having a pair of lane markings, it is easy to reconstruct the continuous lane markings based on the identified lane markings, as shown in Figure 16b,c,e,f. However, for images having only one lane marking, it is a challenge to determine the exact location of the other one lane marking, and two variables, namely lane marking location in previous image and lane width are proposed to solve this problem. Finally, a pair of lane markings for each laser image can be reconstructed, as shown in the right Figure 16h,i.

The lane width depends on the distance between the coordinates of the left and right lane markings. The coordinates of the left and right lane markings for current and previous images are stored in the vectors $X_{l}={\left[x_{l}^{c},x_{l}^{p}\right]}^{T}$, $X_{r}={\left[x_{r}^{c},x_{r}^{p}\right]}^{T}$, respectively. Eventually, a pair of lane marking along traveling direction can be continuously reconstructed with (24) and (25):

(24) $$D_{cp}=\left\{ \begin{array}{l} \left|x_{l}^{c}-x_{l}^{p}\right|\\ \left|x_{r}^{c}-x_{r}^{p}\right| \end{array}\right.,\;D_{cp}\leq T_{os}$$

(25) $$D_{lr}=\left|x_{l}^{c}-x_{r}^{c}\right|,\;D_{lr}\leq T_{w}$$

where *D_cp_* is the offset of left or right lane marking locations between previous and current images; *T_os_* refers to the tolerable range of lane marking offsets; *D_lr_* is the actual lane width; *T_w_* represents the tolerable range of lane widths.

## 4. Case Study

To validate the effectiveness of the new methodology in lane marking identification and localization, a 7500 ft-long asphalt pavement section is chosen as a test bed in this study. Data collection starts at GPS coordinate of 34.8681, −92.401996, and ends at the GPS coordinate of 34.881418, −92.39309, located at 17468 to 16420 Maumelle Blvd. in Maumelle, AR, USA. The test section consists of 1000 laser images, and each image may either contain or not contain lane marking. In this study the binarization, identification, and localization of lane markings are validated.

### 4.1. Binarization Result Analysis

To quantitatively describe binarization results of lane marking, three evaluation metrics namely precision, recall, and F-score are introduced. For each lane marking, it can be regarded as “True Positive (TP)” if the automatic binarization result exactly matches with the manual survey result (ground truth); otherwise, it would be considered as the “False Negative (FN)”. For non-lane marking, it can be considered as “True Negative (TN)” if the binarized non-lane marking still is non-lane marking; otherwise, it would be considered as the “False Positive (FP)”. In this study TP and TN are regarded as the acceptable binarization results, while FP and FN are considered as the unacceptable binarization results.

Once the TP, TN, FP, and FN are determined, three evaluation metrics can be calculated, as described in Equations (26)–(28). Generally, the larger the evaluation metrics is, the better the performance of the test algorithm is [54]. An ideal or robust algorithm would have values of all evaluation metric approximating to one:

(26) Precision=TP/(TP+FP)

(27) Recall=TP/(TP+FN)

(28) $$F=\frac{2\times Precision\times Recall}{Precision+Recall}$$

Several methods, the namely OTSU threshold method [46], minimum threshold method [47], Yen’s method [55], Li’s cross entropy method [56], ISODATA method [57], and the new method are used to verify the binarization effects, as summarized in Table 2.

Note that the new method produces the best binarization results when compared with the other five binarization methods, with a precision of 0.97, recall of 0.96, and F-measure of 0.96, followed by is the OTSU threshold method, minimum threshold method, ISODATA method, Yen’s method, and Li’s cross entropy method. Therefore, it can be concluded that the new binarization method is robust for 2D laser image binarization in this calculation example.

### 4.2. Identification and Reconstruction Result Analysis

**Figure 17:**
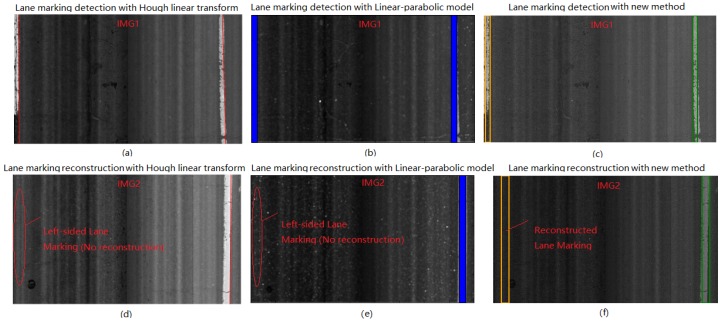
Comparison of detection and reconstruction results of lane marking with: (**a**,**d**) Hough linear transform; (**c**,**e**) linear-parabolic method; (**d**,**f**) the new method.

To validate the effects of the new method on road lane marking detection, the detection result from the new method is compared with that from two widely used methods, namely the Hough linear transform and linear-parabolic lane method. The laser image has a size of 2048 × 3604 pixels. Two laser images are chosen to demonstrate the implementation of lane marking detection and reconstruction. The colorful lines and solid rectangles of IMG1 in Figure 17a–c show the lane marking detection results based on the three methods. For the lane marking reconstruction, both Hough linear transform and linear-parabolic method cannot successfully reconstruct the dash lane marking in IMG2, as shown in Figure 17d,e, however, the new method can efficiently reconstruct the dash lane marking, as shown in Figure 17f.

In this study the precision, recall, and F-measure are used to evaluate the effects of three methods on lane marking detection. The lane marking detection accuracy with the three methods are given in Table 3. It can be observed that the new method produces the best detection result among them, with a precision of 0.95, recall of 0.93, and F-measure of 0.94, based on 1000 test laser images, followed by the linear-parabolic lane method which produces a detection result with a precision of 0.91, recall of 0.89, and F-measure of 0.90. The Hough linear transform produces a result with a F-measure of 0.88. The corresponding results based on the three methods are given in Table 3.

The three methods are implemented using Python & OpenCV running on an Intel(R) Core(TM) i7-7700K @4.2 GHz computer. The processing times for the three methods are given in Table 3. With the new method, the processing times for image binarization, candidate lane marking determination, and true road lane marking detection and reconstruction are 1.263, 0.156 s, and 0.004 s, respectively. The total processing time is about 1.423 s per frame, which is slightly longer than that of the other two methods. Therefore, the new method is not suitable for real-time processing of lane marking detection and is recommended to be used for image post-processing with the purpose of pavement performance evaluation.

In addition, a precision of 0.95, recall of 0.91, and F-measure of 0.94 are obtained for the lane marking reconstruction results based on 1000 test laser images. It can be concluded that the new method is robust for lane marking detection and reconstruction. The exact identification and localization of lane marking are crucial for pavement lane-based study, such as crack detection and classification, rutting measurement and evaluation, etc.

## 5. Conclusions and Recommendations

In this paper a new methodology is proposed to detect and locate road lane markings with 2D laser images collected from a DHDV. Firstly, the multi-box segmentation-based traversal method to binarize 2D laser images is presented, and excellent binarization results are produced when compared with other methods such as the OTSU method, minimum method, ISODATA method, Yen’s method, and Li’s cross entropy method, with a precision of 0.97, and recall of 0.96. Subsequently the morphological closing method and marching square method are employed to determine the contours of the potential lane markings, where generally one contouring box represents one candidate lane marking. Thirdly, a linear support vector machine is used to distinguish true lane markings from candidate lane markings based on contour box attributes, with a precision of 0.95, recall of 0.93, and F-measure of 0.94. The new method produces the better detection results when compared with the Hough linear transform and linear-parabolic lane methods. Finally, the continuous true lane markings along the traveling direction are reconstructed with the location of adjacent lane markings and road lane width. The findings indicate that the proposed methodology is robust for the detection and location of road lane markings in 2D laser images, which would benefit in road lane-based pavement distress measurement and evaluation, such as pavement cracking detection and classification, rutting measurement and so on.

Although LSVM based on contour box attributes can efficiently separate out true lane markings from fake targets, the effects of pedestrian crosswalks and lane direction arrows on lane marking identification cannot be avoided. As a future improvement, a new strategy could be developed to solve this issue, and simultaneously examine lane-based crack detection and classification.

## Figures and Tables

**Table 1:** Summary of Contour Box Attributes.

IMG ID	Box ID	Box Width	Box Height	Contour Length	Contour Complexity	∇x	∇y	$I_{t}(\%)$
IMG1	ID1	170	2019	4310	1.0	0.004	0.012	98.8
IMG2	ID1	152	1149	2630	1.0	0.008	0.015	98.4
	ID2	151	2019	4314	1.0	0.004	0.013	98.6
	ID3	34	34	96	0.7	0.057	0.054	92.2
	ID4	36	61	161	0.8	0.049	0.054	92.6
	ID5	5	3	13	0.8	0.533	0.267	40.4
	ID6	9	10	27	0.7	0.178	0.222	71.5
	ID7	5	6	16	0.7	0.267	0.333	57.3
	ID8	3	5	12	0.8	0.267	0.533	40.4

**Table 2:** Comparison of Binarization Results with Various Methods.

Binarization Methods	# of IMGs	Precision	Recall	F-Measure
OTSU	1000	0.873	0.898	0.885
Minimum	1000	0.866	0.842	0.854
Yen’s Method	1000	0.553	0.900	0.685
Li’s Method	1000	0.644	0.732	0.685
ISODATA	1000	0.813	0.843	0.828
New Method	1000	0.967	0.961	0.964

**Table 3:** Comparison of Lane Marking Identification Results with Various Methods.

Detection Methods	# of IMGs	Computing Time(s)/Frame	Precision	Recall	F-Measure
Hough Linear Transform	1000	1.135	0.91	0.86	0.88
Linear-parabolic Lane Method	1000	1.124	0.91	0.89	0.90
Newly Proposed Method	1000	1.423	0.95	0.93	0.94
